# Fracture of a Polyethylene Tibial Post in a Scorpio Posterior-Stabilized Knee Prosthesis

**DOI:** 10.4055/cios.2009.1.2.118

**Published:** 2009-05-27

**Authors:** Hong Chul Lim, Ji Hoon Bae, Jin Ho Hwang, Seung Joo Kim, Ji Yeol Yoon

**Affiliations:** Department of Orthopedic Surgery, Korea University College of Medicine, Guro Hospital, Seoul, Korea.

**Keywords:** Total knee arthroplasty, Posterior-stabilized, Anterior impingement, Polyethylene wear, Post fracture

## Abstract

We report the case of a polyethylene tibial post fracture in a 72-year-old woman 14 months after a Scorpio posterior-stabilized (PS) total knee arthroplasty. The polyethylene wear was found around the fracture site of the post, especially over the anterior aspect of the post base. The failure mechanism of the post fracture in the present case was anterior impingement with excessive wear over the base of the anterior aspect of the tibial post, which became a stress-riser of post and cam articulation. This is the first report of a polyethylene tibial post fracture of a Scorpio PS prosthesis.

Posterior-stabilized (PS) knee prostheses have been used extensively in total knee arthroplasties (TKAs) with excellent long-term results.^1^,2) The key feature of these prostheses is the femoral cam and tibial post mechanism that limits posterior displacement and produces femoral rollback. As a mechanical restraint against posterior tibial subluxations, the polyethylene tibial post is susceptible to damage related to impingement, wear, and breakage.^3^,4) Tibial post failures, resulting in intermittent pain and instability, have been reported in PS-TKAs.^5^-7) We report the case of a patient who presented with acute pain and swelling following a TKA caused by a polyethylene tibial post fracture.

## CASE REPORT

A 72-year-old woman with osteoarthritis of both knees underwent a left TKA in March 2005. A standard midline anterior incision with a medial arthrotomy was used to implant a Scorpio (Stryker, Mahwah, NJ, USA) PS knee prosthesis. Preoperatively, the radiographs showed a 4° varus deformity of the right knee and a 7° varus deformity of the left knee. The range of motion of both knees was 5° to 135°. There was weakness of the left thigh flexors (motor grade IV) because the patient suffered from a cerebrovascular accident 10 years earlier. During surgery, an 8 mm polyethylene tibial insert was used, which provided good stability in full extension, mid-flexion, and full flexion of the left knee intraoperatively. The patella was not replaced. Postoperatively, the patient recovered well, with knee flexion from 0° to 125° at 6 weeks.

**Fig. 1:**
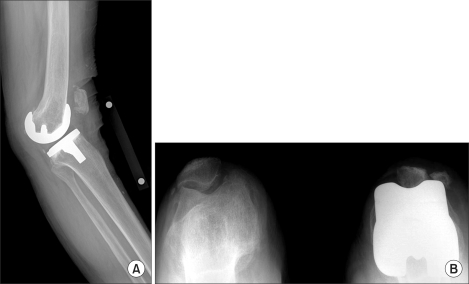
Radiographs taken in the emergency department 14 months after total knee arthroplasty following slipping and falling. (A) Lateral radiograph showing genu recurvatum, (B) 45° Sky line view showing vertical fracture of the left patella.

**Fig. 2:**
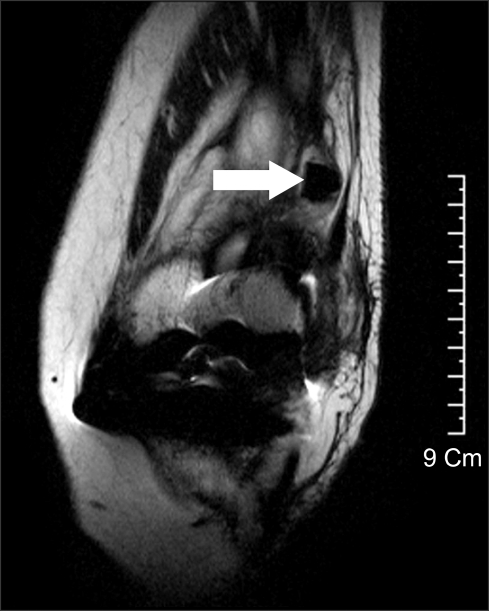
Coronal fast-spin-echo magnetic resonance image of the left knee in a patient following a slip and fall revealed a loose body in the suprapatellar pouch of the knee consistent in shape with the tip of the tibial polyethylene post.

**Fig. 3:**
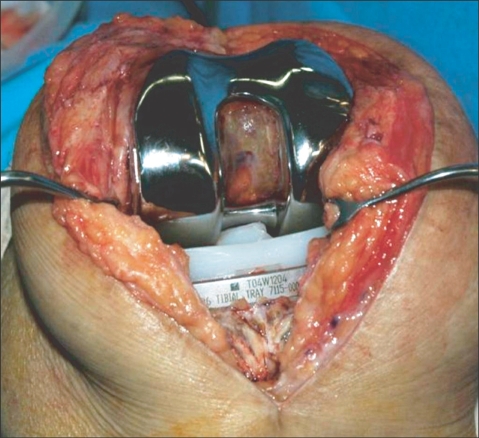
Intraoperative photograph showing a fracture of the polyethylene tibial post at its base.

**Fig. 4:**
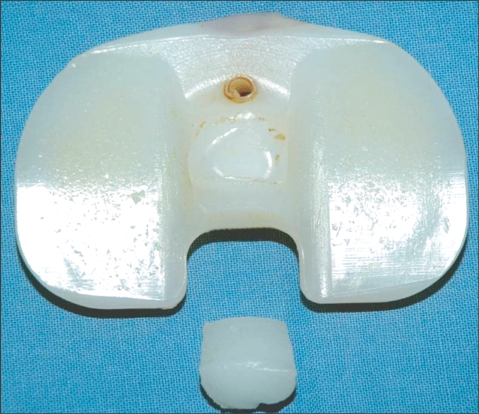
A photograph of the retrieved insert showing fracture of the poly-ethylene tibial post at its base.

In May 2006, 14 months after the TKA, the patient presented with painful swelling and an effusion of the left knee after slipping and falling. Radiographs showed a vertical fracture of the lateral aspect of the patella and genu recurvatum (Fig. 1). The patellar fracture was treated conservatively. Examination of her left knee revealed genu recurvatum and soft end points on varus and valgus stress testing. Soft tissue and ligamentous injuries were suspected. Magnetic resonance imaging (MRI) was obtained to evaluate an acute ligamentous injury. The MRI demonstrated a loose body fragment in the suprapatellar pouch (Fig. 2). Based on the shape and signal density, the loose body was believed to be a part of the post from the polyethylene tibial insert. Arthroscopy revealed a post fracture as the source of the loose body and a broken polyethylene fragment was found in the suprapatellar pouch. The tibial post was broken at its base with considerable wear of the anterior aspect (Figs. 3 and 4).

Revision of polyethylene tibial insert was performed 1 month after the arthroscopic procedure. Because the prosthesis appeared well-fixed and in good position, and there was no significant articular surface wear or asymmetry of the thickness of the two condyles, the broken polyethylene tibial insert alone was replaced with a thicker polyethylene tibial insert (12 mm). Correct alignment and stability was achieved after replacement of the polyethylene insert. The patellar fracture was treated conservatively. She had an uneventful recovery. One month postoperatively the knee was stable on examination and motion was 0° to 120°.

Three months after replacement of the polyethylene insert, the patient complained of pain over the anterolateral aspect of the patella. A radiograph showed a small lateral fragment of the patella non-union. The patient was advised to have the small fragment of the patella excised and to undergo arthroscopic surgery to confirm the source of the pain. The small fragment of the patella non-union was excised and arthroscopy was performed. During the surgical procedure, polyethylene wear over the anterior aspect of the base of the post was noted only 3 months after the polyethylene exchange. There was no significant instability at that time, and no further procedures were performed. The patient was informed of a possible revision of the femoral and tibial components. Two years postoperatively, the patient had no complaints of pain or instability.

## DISCUSSION

The failure mechanism of the post fracture in the present case was anterior impingement with excessive wear over the base of the anterior aspect of the tibial post, which became a stress-riser of post and cam articulation. When the lift-off force overcame the resistance of post integrity, the fracture occurred at the posterior base and propagated anteriorly.7) Our hypothesis is that weakness of thigh flexors due to the cerebrovascular accident caused genu recurvatum, which is one of the causes of anterior tibial impingement. Factors known to cause anterior tibial post impingement include femoral cam and tibial post design that allow hyperextension of the knee, a relatively large extension gap compared with flexion gap, a flexed position of the femoral component relative to the sagittal axis of the knee, and an excessive posterior slope of the tibial component.^3^,^4^,8) Failure to use a sufficiently large polyethylene insert to avoid hyperextension of the knee may also result in anterior impingement of the femoral component on the tibial post, which can lead to wear or fracture. Callaghan et al.3) suggested that the surgeon should avoid flexion of the femoral component and posterior tibial slope of the proximal tibial resection. Also, femoral campost designs for the posterior cruciate substituting knee replacement should allow for relative hyperextension and rotation of the components without femoral camtibial post impingement.

In the present case, a MRI was obtained to evaluate instability when the patient presented with painful swelling and an effusion of the left knee after slipping and falling. A retrospective review of the MRI demonstrated a loose body in the suprapatellar pouch of the knee consistent in shape with the tip of the post. We believe that a high index of suspicion and close scrutiny of low signal density of the tibial polyethylene insert may make the diagnosis possible with MRI.

After the diagnosis has been established in the majority of patients, revision of the polyethylene insert should be considered if the components are well-fixed and in good sagittal and rotational alignment. If the components are loose or malpositioned, complete revision is recommended. Other reports have cautioned that isolated exchange of the polyethylene insert is associated with a high probability of subsequent revision of all components of the total knee.^9^,10) In the present case, polyethylene wear over the anterior aspect of the base of the post occurred only 3 months after a polyethylene exchange during arthroscopy. This finding also resulted from anterior impingement due to a tendency for genu recurvatum. We believe a brace should be considered permanently to prevent genu recurvatum.
